# First report of surra (*Trypanosoma evansi* infection) in a Tunisian dog

**DOI:** 10.1051/parasite/2015004

**Published:** 2015-02-06

**Authors:** Mohamed Ridha Rjeibi, Taoufik Ben Hamida, Zara Dalgatova, Tarek Mahjoub, Ahmed Rejeb, Walid Dridi, Mohamed Gharbi

**Affiliations:** 1 Laboratoire de Parasitologie, Université de la Manouba, École Nationale de Médecine Vétérinaire de Sidi Thabet 2020 Sidi Thabet Tunisia; 2 Laboratoire de Parasitologie, Institut de la Recherche Vétérinaire de Tunisie 20 Rue de Jebel Lakdhar, La Rabta 1006 Tunis Tunisia; 3 Laboratoire d’Anatomie-Pathologique, Université de la Manouba, École Nationale de Médecine Vétérinaire de Sidi Thabet 2020 Sidi Thabet Tunisia

**Keywords:** Surra, Dog, *Trypanosoma evansi*, Molecular identification, Tunisia

## Abstract

*Trypanosoma evansi*, the agent of surra, is a salivarian trypanosome, originating from Africa. Surra is a major disease in camels, equines and dogs, in which it can often be fatal in the absence of treatment. Animals exhibit nonspecific clinical signs (anaemia, loss of weight and abortion). In the present survey, a blood sample was collected in Sousse (Central Tunisia) from a dog that presented clinical signs of trypanosomiasis. Giemsa-stained blood smears and PCR were performed. ITS1 sequences from blood had 99.8 and 99.5% homology with published *T. evansi* sequences from cattle and camels, respectively*.* To our knowledge, this is the first report of *T. evansi* in a Tunisian dog.

## Abstract (fr)

*Trypanosoma evansi*, l’agent du surra, est un trypanosome salivaria d’origine africaine. Le surra est une maladie importante chez le dromadaire, les équidés et le chien, qui est souvent fatale en l’absence de traitement. L’animal présente des signes cliniques non spécifiques (anémie, perte de poids, avortement). Dans la présente étude, l’échantillon de sang a été prélevé à Sousse (centre de la Tunisie) d’un chien présentant des signes cliniques de trypanosomose. Des étalements de sang colorés au Giemsa et des PCR ont été réalisés. Les séquences ITS1 avaient 99,8 et 99,5 % d’homologie avec des séquences de *T. evansi*, respectivement de bovins et de dromadaires. A notre connaissance, ceci est la première mention de *T. evansi* chez un chien en Tunisie.

## Introduction

*Trypanosoma evansi* is the most widely distributed pathogenic salivarian trypanosome in animals, it causes a significant disease called surra. Main vectors worldwide are tabanids and *Stomoxys* spp.; oral transmission has been reported in a very wide range of domestic and wild hosts. Since 2008, notification of surra became compulsory not only in horses, because it is now considered a multi-species disease by the World Animal Health Organization (OIE) [13, 15]. This parasite is widely distributed; it is present in North Africa, in the Middle East, Turkey, India, up to 53° North in Russia, across all South-East Asia, down to Indonesia and the Philippines. This parasite was introduced into Latin America by the conquistadores [5]. *Trypanosoma evansi* has been reported in the Canary Islands (Spain) where it has been regularly observed since 1995 [8], and the Spanish mainland (Alicante Province) in a mixed camel and horse farms [16]. In France, a single outbreak occurred in camels imported from the Canary Islands [4]. In Africa, *T. evansi* is mainly a parasite of camels, which represents both the main host and reservoir. *T. evansi* can infect cattle (*Bos taurus*) [6], pigs (*Sus scrofa*), domestic sheep (*Ovis aries*) and goats (*Capra hircus*) [5]. It is considered as non-pathogenic for African buffalo (*Syncerus caffer*) [14] and is occasionally reported in horses, dogs and cats [5, 18]. Since 2005, surra has been considered as zoonotic, after the discovery of human clinical cases in India and Egypt [9, 10]. In this paper, we report the first case of surra in North Africa in a Tunisian dog.

## Materials and methods

### Case report

A two-year-old female Pit-bull dog, living in a tourist leisure centre with 25 dromedaries in the region of Sousse (Central Tunisia) presented a history of chronic ocular symptoms, hyporexia and emaciation. Leishmaniasis was suspected and both lymph node and blood smears were Giemsa stained and examined under a microscope at ×1000 magnification with immersion oil.

### Genetic analyses

The DNA was extracted from the blood sample using a Genomic DNA Purification Kit (Promega, Madison, USA). *Trypanosoma evansi* PCR was performed with a set of primers that amplifies a 480 bp region of *T. evansi* ITS1 rDNA gene [12]. The forward primer was ITS1 CF (5′-CCGGAAGTTCACCGATATTG-3′) and the reverse primer was ITS1 BR (5′-TGCTGCGTTCTTCAACGAA-3′). The PCR mixture consisted of 2.5 μL of 10× PCR buffer (50 mM Tris-HCl; pH 8.5; 50 mM NaCl), 2 mM MgCl_2_, 0.2 mM of each dNTP, 0.2 μM of each primer, 0.5 U Taq Polymerase (Vivantis, Chino, California), 3 μL of DNA template and distilled water to a total volume of 25 μL. The DNA was amplified using the following programme: 5 min denaturation at 94 °C, followed by 35 cycles (94 °C for 40 s, 58 °C for 40 s and 72 °C for 90 s) and a final extension at 72 °C for 5 min. The PCR product was purified with the Wizard SV Gel and PCR Clean-Up System (Promega, Madison, USA) according to the manufacturer’s instructions. The fragment was sequenced in both directions, using the same primers as for PCR. A conventional BigDye Terminator Cycle Sequencing Ready Reaction Kit (Applied Biosystems, Foster City, CA) and an ABI3730XL automated DNA sequencer were used. The chromatograms were evaluated with ChromasPro software (version 1.7.4). The MEGA 5.1 software program was used to perform multiple sequence alignments [17]. The sequences were compared with the GenBank database by nucleotide sequence homology. Searches were made at the network server of the National Center for Biotechnology Information (NCBI) using BLAST.

## Results

**Figure 1.**
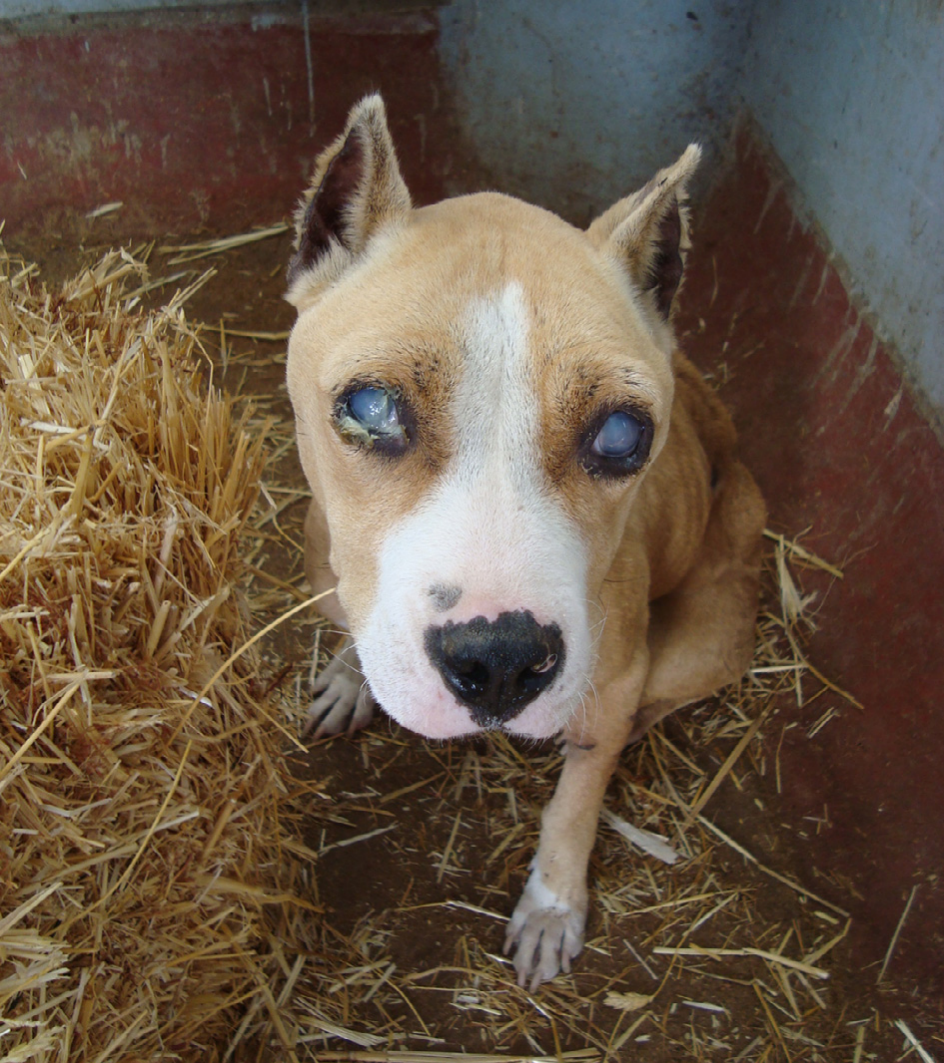
Bilateral purulent blue keratitis in a dog with surra.

**Figure 2.**
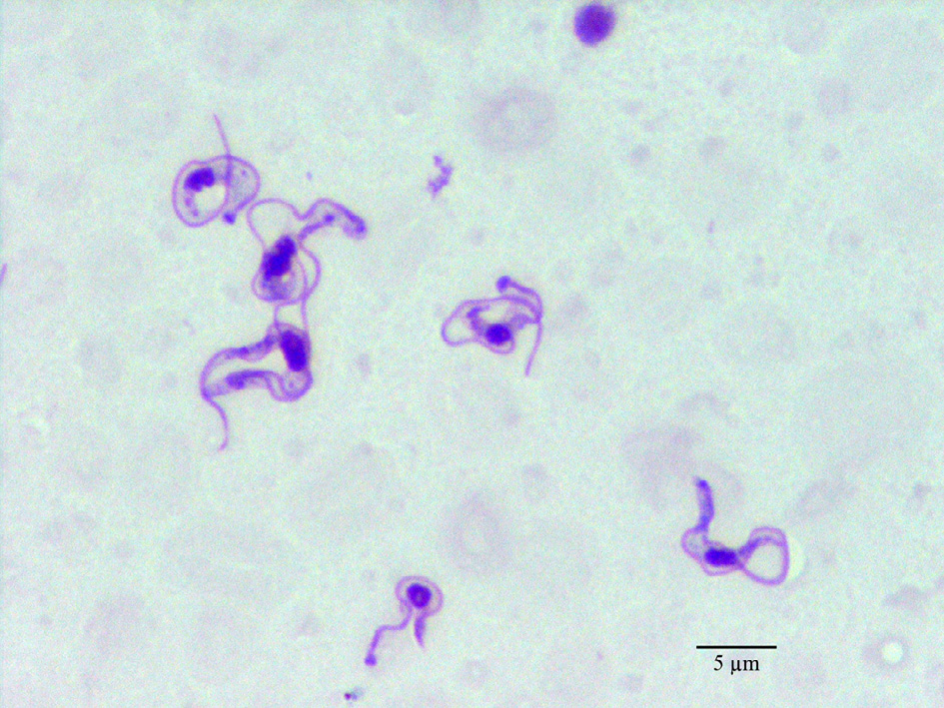
*Trypanosoma evansi*, dog, Giemsa-stained blood smear.

The clinical examination showed significant muscular emaciation and bilateral keratitis with corneal opacity and impaired eyesight (Fig. 1). The lymph nodes were not enlarged but, due to the high prevalence of canine leishmaniasis [2] and babesiosis [11] in Tunisia, a blood smear and a lymph node biopsy were performed, they were negative for *Babesia* spp. and *Leishmania infantum* but showed high population of *Trypanosoma* spp. (Fig. 2). The dog presented hypoglycaemia (0.76 g/L), uraemia (0.9 g/L), hyperproteinaemia (84 g/L) and normocytic (71.7 fl) normochromic (31.6 g/dL) regenerative anaemia (6.8 g/dL) with severe thrombocytopenia (5 × 10^3^/μL) (Table 1).

**Table 1.** Results of biochemical and haematological analyses of the dog infected with *Trypanosoma evansi.*

	Subject’s values	Reference values
**Biochemical results**		
Glycaemia (g/L)	0.76	0.6–1.2
Urea (g/L)	0.9	0.2–0.5
Creatininaemia (mg/L)	8.0	10–20
ALT (U/L)	68.0	21–102
AST (U/L)	69.0	23–66
GGT (U/L)	<4	<10
Total Protein (g/L)	84.0	54–71
**Haematological results**		
Leucocytes (10^9^/L)	9.41	6–17
Erythrocytes (10^12^/L)	3	5.5-8.5
Corpuscular haemoglobin (g/dL)	6.8	12–18
Haematocrit (%)	21.5	37–55
Mean corpuscular volume (fl)	71.7	60–77
Mean corpuscular haemoglobin levels (pg)	22.7	19.5–24.5
Mean corpuscular haemoglobin concentration (g/dL)	31.6	32–36
Platelets (10^9^/L)	5	200–500
IDR SV (fl)	42.6	26.3–38.5
IDR CV (%)	16.9	9.2–12
Monocytes (10^9^/L)	0.66	0.15–1.35
Eosinophils (10^9^/L)	0	0.1–1.25
Basophils (10^9^/L)	0.01	Rare

The dog was treated with intramuscular diminazene aceturate at the conventional dose of 5 mg/kg but died 2 weeks later. The necropsy revealed cachexia, severe anaemia, subcutaneous oedema, acute interstitial hepatitis and nephritis. The dog also presented congestive inconspicuous generalized adenitis, gastroenteritis with congestive colitis, bilateral mucopurulent conjunctivitis and significant splenomegaly with hyperplasia of the red pulp.

A novel *T. evansi* ITS1 rDNA genotype named TETND01 (GenBank Accession Number KJ741365) was identified in this study.

The BLAST comparison of the partial sequences of the ITS1 rDNA gene revealed 99.8% homology between our isolate (KJ741365) and isolates from cattle (AY912277) in Thailand, 99.5% homology with isolates from dromedaries (AB551922) from Egypt and from Thai deer (AY912279), 99.1% homology with isolates from Thai buffalo (AY912270) and from Chinese mules (FJ712712).

## Discussion

Trypanosomiasis is a common protozoan infection in camels in Tunisia; its seroprevalence was estimated to be 18% by Gallo et al. in 1989 [7]. *Trypanosoma evansi* is reported in North Africa, Southern Europe, Latin America and Asia [13]. It is commonly pathogenic in camels, horses, cattle and occasionally in humans [9, 10] and dogs [5]. In humans, innate immunity against *T. evansi* could depend on a plasmatic trypanolytic factor, namely apolipoprotein L-1. Indeed, a deficit in both apolipoprotein L-1 alleles has been discovered in an Indian patient infected by *T. evansi* [19]. *T. evansi* in dogs is not frequent; two cases have been reported: one in Germany [3] and another in Afghanistan [1]. Lethargy, weight loss and ocular lesions seem to be constant symptoms in canine surra [1, 3]. Unlike Aref et al. in 2013 [1], we observe neither diarrhoea nor cardiac disease in the present case. Moreover, Aref et al. did not report any haematological changes since they found normal cell packed volumes and total white cell values. The contamination could occur either orally (by ingestion of aborted placenta or foetuses eliminated by infected females) or by several haematophagous vector species.

Veterinarians in non-endemic regions such as Europe should consider this disease in dogs with a history of living in endemic countries such as Tunisia and presenting weight loss and ocular involvement. The differential diagnosis should be established with canine leishmaniasis (including atypical forms with no obvious lymph node enlargement) and babesiosis. Further studies are needed to estimate the prevalence of different *T. evansi* infection forms (carrier or clinical forms) in Tunisian dogs.
